# The Relevance and Implications of Monoclonal Antibody Therapies on Traumatic Brain Injury Pathologies

**DOI:** 10.3390/biomedicines12122698

**Published:** 2024-11-26

**Authors:** Ping Wang, Starlyn Okada-Rising, Anke H. Scultetus, Zachary S. Bailey

**Affiliations:** Brain Trauma Neuroprotection, Walter Reed Army Institute of Research, Silver Spring, MD 20910, USA; starlyn.l.okada-rising.civ@health.mil (S.O.-R.); anke.h.scultetus2.civ@health.mil (A.H.S.); zbailey3@gmail.com (Z.S.B.)

**Keywords:** traumatic brain injury (TBI), monoclonal antibodies (mAbs), monoclonal antibody therapy, secondary injury cascades, pathological TBI processes, oxidative stress, coagulopathy, neuroinflammation, neurodegeneration, neurotoxicity, blood–brain barrier (BBB) dysfunction, neurological diseases

## Abstract

Traumatic brain injury (TBI) is a global public health concern. It remains one of the leading causes of morbidity and mortality. TBI pathology involves complex secondary injury cascades that are associated with cellular and molecular dysfunction, including oxidative stress, coagulopathy, neuroinflammation, neurodegeneration, neurotoxicity, and blood–brain barrier (BBB) dysfunction, among others. These pathological processes manifest as a diverse array of clinical impairments. They serve as targets for potential therapeutic intervention not only in TBI but also in other diseases. Monoclonal antibodies (mAbs) have been used as key therapeutic agents targeting these mechanisms for the treatment of diverse diseases, including neurological diseases such as Alzheimer’s disease (AD). MAb therapies provide a tool to block disease pathways with target specificity that may be capable of mitigating the secondary injury cascades following TBI. This article reviews the pathophysiology of TBI and the molecular mechanisms of action of mAbs that target these shared pathological pathways in a wide range of diseases. Publicly available databases for various applications of mAb therapy were searched and further classified to assess relevance to TBI pathology and evaluate current stages of development. The authors intend for this review to highlight the potential impact of current mAb technology within pathological TBI processes.

## 1. Introduction

Traumatic brain injury (TBI) is a public health concern with a prevalence of about 69 million worldwide per year [1]. In the U.S. alone, about 1.7 million TBIs are reported annually, which includes a range of mild to severe brain injuries [2,3]. Millions of TBI-related visits to an emergency department (ED), hospitalizations, or deaths occur annually. TBI patients can experience short-term or permanent disabilities in multiple domains of functioning, including cognition, physical health, psychological health, and social life. TBI imposes significant health, social, and economic burdens. Various therapeutic interventions are being studied in pre-clinical animal models of TBI, but effective clinical therapeutic treatments for TBI recovery are still lacking [4,5,6,7,8,9,10,11]. This problem is likely to be augmented as the TBI survivor population continues to increase [12].

Mechanistically, TBI pathology manifests from two primary mechanisms: primary and secondary damage. Primary injury occurs at the moment of initial trauma and can include skull fracture, cerebral hemorrhage, and diffuse axonal injury [4,13]. These primary mechanisms elicit complex secondary injury mechanisms that drive subsequent pathology and include cellular and molecular dysfunction, including oxidative stress, neuroinflammation, neurodegeneration, neurotoxicity, and blood–brain barrier (BBB) dysfunction, among others [4]. These pathological processes manifest as a diverse array of clinical impairments and serve as targets for potential therapeutic intervention [4,6].

TBI-induced cerebral injury is a mixture of structural, cellular, and vascular injury. Current therapeutic interventions involve neuroprotection, neurovascular regeneration, and neurorestoration for treating TBI. These strategies often target a single factor-mediating secondary injury. These proclaimed specific factors often have additional and unexpected effects on other pathways. Monoclonal antibodies (mAbs) can bind to the cell or pathway-specific molecules and modulate injury mechanisms. MAb therapies provide a tool to influence specific cellular and molecular pathways that may be capable of mitigating the secondary injury cascades following TBI and offering a more precision medicine approach [14].

Antibodies (or immunoglobulins) are naturally occurring protective proteins that offer one of the body’s main lines of defense against foreign substances. Antibodies are Y-shaped proteins. They are composed of two heavy chains and two light chains. Each chain contains a variable region and a constant region. The variable region contains a sequence of amino acids that gives each antibody its specificity towards a particular antigen and even a particular binding site on that antigen, in the case of monoclonal antibodies. This specificity maximizes the potential for harnessing monoclonal antibodies as a therapeutic strategy by inherently reducing cross-reactivity within biological systems [15].

**Figure 1:**
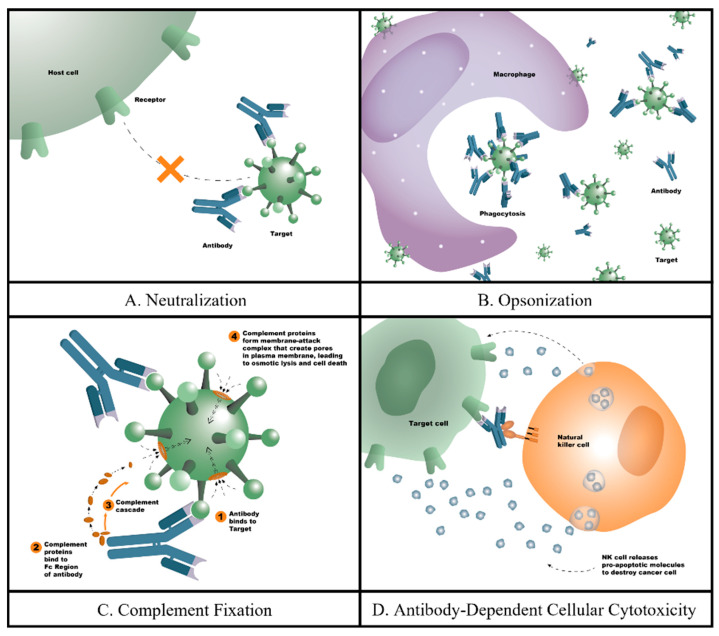
Four main mechanisms of monoclonal antibodies: (**A**) neutralization: this process can occur by allosteric inhibition, in which antibodies binding to the surface of the antigen prevent the antigen from reaching and interacting with the target cells; (**B**) opsonization: a process by which pathogens are coated with antibodies to increase their susceptibility for phagocytosis; (**C**) complement fixation: an immune reaction by which antibody binding to the pathogen can recruit and activate the complement system and facilitate the recruitment of phagocytic cells; (**D**) antibody-dependent cellular cytotoxicity: a process by which antibodies attach to target cells and recruit effector cells to induce target cell death.

Antibodies can defend against foreign substances through various mechanisms [16,17] (Figure 1). In some cases, antibody removal of foreign substances can be achieved without the recruitment of other molecular/cellular support. Neutralization is a process by which antibodies binding to the surface of the antigen prevent the antigen from reaching and interacting with the target cell(s) [17,18]. Alternatively, antibody binding can create aggregates by cross-linking pathogens through the use of the multiple binding sites available on the antibody. These aggregates can subsequently be more efficiently filtered from the body through the kidneys or spleen. Other mechanisms of action involve the recruitment of cellular or molecular support to remove the pathogen. Antibody binding to the pathogen can recruit and activate the complement system, facilitate the recruitment of phagocytic cells, or recruit natural killer cells, which secrete cytotoxins to kill the pathogen (antibody-dependent cell-mediated cytotoxicity; ADCC) [16,17].

**Figure 2:**
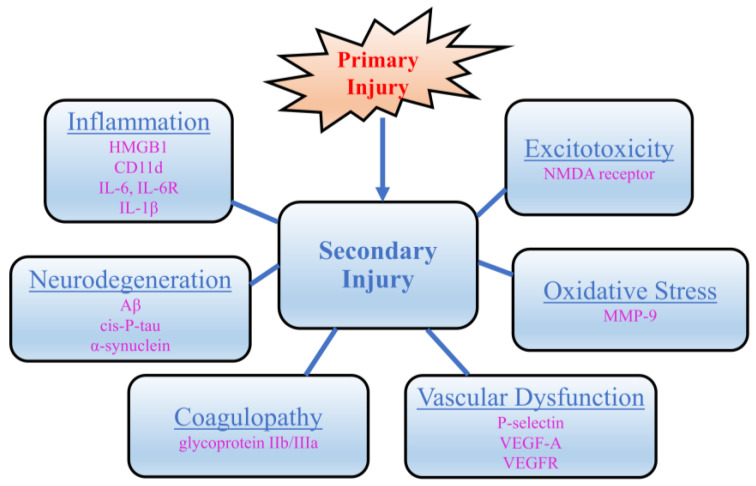
Secondary injury pathologies in TBI and previously studied therapeutic targets. The several key areas include inflammation (targets: high mobility group box 1 (HMGB1), cluster of differentiation 11d (CD11d), interleukin 6 (IL-6), interleukin 6 receptor (IL-6R), and interleukin 1 beta (IL-1β)), vascular function (targets: P-selectin, vascular endothelial growth factors-a (VEGF-A), vascular endothelial growth factors receptor (VEGFR)), coagulation (targets: glycoprotein IIb/IIIa), excitotoxicity (targets: N-methyl-D-aspartate receptor (NMDA) receptor), oxidative stress (targets: matrix metalloproteinase-9 (MMP-9)), and neurodegeneration (targets: amyloid beta (Aβ), cis-P-tau, and α-synuclein).

Over the years, scientists have taken advantage of the natural function of antibodies in their pursuit of finding treatments for a wide range of diseases, such as many types of cancer, as well as autoimmune, infectious, and hematological diseases [19]. Hybridoma technology gave rise to the mass production of murine antibodies [20,21] and, later, chimeric and humanized antibodies [22]. The development of humanized mAbs helped launch mAbs into the commercial market through the discovery of the complementary-determining region grafting technique, which allowed for even greater conservation of human-derived regions [23]. Humanized mAbs have a human origin for the constant regions and for most of the variable regions—only the hypervariable complementary-determining regions are transplanted from a non-human source [23]. The FDA approved the first mAb for therapeutic use in 1986 [24]. Since then, the market has experienced dramatic growth. Globally, commercial companies have studied over 570 mAbs in clinical trials [25]. Over 79 mAbs have been approved for therapeutic use by the U.S. FDA over the years [19,21]. Major technological advancements in discovery and development technologies have resulted in augmented market growth and mAbs available for therapeutic use [19,26,27,28,29]. Despite the growth, few studies have evaluated mAb potential within the TBI pathology. The authors intend for this review to highlight the potential impact and evaluate the possibilities for current mAb therapeutic technology as a relevant tool for the treatment of secondary TBI injury. To develop a firm understanding of the state of the science and literature landscape, we reviewed publicly available databases (Google Scholar, PubMed, Clinicaltrials.gov, etc.) for various applications of mAb therapy. The results were further classified to assess relevance to TBI pathology and evaluate current stages of development. A number of factors contribute to secondary TBI injury. While others exist, this work identified and focused on several key areas of relevance, including inflammation, vascular function, coagulation, excitotoxicity, oxidative stress, and neurodegeneration (Table 1, Figure 2).

## 2. mAbs and Inflammation

### 2.1. Pathophysiology Relevance

Primary damage resulting from TBI triggers a complex and heterogeneous neuroinflammatory response. This response involves various cytokines, chemokines, and inflammatory molecules that coordinate the functions of microglia, astrocytes, and infiltrating immune cells [62,63]. During the acute stages after injury, these processes are beneficial for mitigating injury progression, repairing damaged cells, and protecting against pathogen infiltration [64,65,66,67,68]. However, when the inflammatory response becomes persistent and unregulated, it can hinder recovery and exacerbate oxidative stress, cell death, and neurodegeneration [69,70]. As such, chronic inflammation has historically been a focus of therapeutic intervention strategies.

Upon injury, damage-associated mediator proteins, including high-mobility group protein B1 (HMGB1), are passively released from injured cells [71,72]. This release is continued by secondary injury mechanisms that create a detrimental feedback loop that leads to robust production of pro-inflammatory cytokines, including tumor necrosis factor-alpha (TNF-α), interleukin 6 (IL-6), interleukin 1 beta (IL-1β), and interferon-gamma (IFN- γ) [72,73]. The expression of these pro-inflammatory mediators initiates several downstream processes, including the increased permeability of the BBB and the upregulation of adhesion molecules. Together, these changes facilitate the infiltration of neutrophils, monocytes, and lymphocytes to cross the BBB into the brain parenchyma [74].

Once these immune cells infiltrate, they contribute to the production of inflammatory mediators and can recruit microglia, the resident immune cells of the brain. Microglia arise from macrophages and can take on a pro-inflammatory and anti-inflammatory phenotype, serving as the first line of defense to the injured brain. Studies have shown that microglia tend to favor the pro-inflammatory phenotype in the acute stages following injury, which aids initial recovery by scavenging cellular debris and coordinating restorative processes. Many of these actions are achieved by the upregulation of pro-inflammatory mediators, neurotoxic chemicals, and free radicals. While the neurotoxic chemicals and free radicals initiate cell death mechanisms, the pro-inflammatory mediators can further propagate the inflammatory response [75].

Astrocytes also play an important role following TBI [76]. They have been shown to promote axonal repair, cell proliferation, neuronal survival, and the inhibition of apoptosis. Additionally, astrocytes are involved in repairing the BBB after injury and can limit cell infiltration and blood component extravasation [77]. However, like microglia, excessive activation has been shown to be detrimental [77]. Astrocyte activation and proliferation are often associated with glial scar formation at the injury site. The scar establishes a barrier around the damaged tissue, separating healthy brain areas from neurotoxic or potentially harmful regions through the production of an inhibitory cellular matrix. While the scar can help contain the damage response, it may also compromise the repair mechanisms of the damaged tissue and hinder the reduction in glial scarring that has been associated with positive outcomes [76].

### 2.2. mAb and TBI Inflammatory Processes: FDA-Approved mAbs and Pre-Approval Testing Phases

Because inflammatory processes are not unique to specific pathologies, many of these mechanisms have been extensively studied in other diseases. mAbs have been utilized against a wide range of non-TBI diseases, particularly those characterized by persistent inflammation, such as irritable bowel (IBD), Crohn’s disease (CD), and rheumatoid arthritis [19]. As previously mentioned, IL-6R, IL-1β, TNF-α, IFN-γ, and HMGB1 have been implicated in TBI pathologies. These same mediators have also been associated with other somatic pathologies, leading to the development of monoclonal antibodies targeting these cytokines or their receptors, many of which have received FDA approval and demonstrate considerable efficacy. Often, multiple mAbs may be indicated for the same pathology, reflecting a variety of mechanisms of action or differences in the construction of the mAb itself [19].

### 2.3. Pre-Approval mAbs

#### 2.3.1. High Mobility Group Box 1 (HMGB1)

High mobility group box 1 (HMGB1) is a significant mediator of injury-induced inflammation, making it a promising target for neuroinflammation to inhibit secondary damage post-TBI. HMGB1 is a pro-inflammatory-like cytokine released due to the activation of other cytokines and passively during cell death. It acts as a nuclear factor that enhances transcription and mediates responses to infection, inflammation, and injury. During TBI, HMGB1 mediates neuroinflammation through the activation of cytokines such as TNF-α and interleukin-1 (IL-1). The over-expression of HMGB1 has been observed in TBI [78] and other neuroinflammatory conditions, including Alzheimer’s disease [79], Parkinson’s disease [80], and subarachnoid hemorrhage [81]. In TBI, HMGB1 is released through the N-methyl-D-aspartate receptor [82]. Pre-clinical studies in rats have shown that anti-HMGB1 antibodies can reduce the accumulation of activated microglia in the cortex of the ipsilateral hemisphere after TBI and prevent neuronal death in the hippocampus [30]. Another rat study model of intracerebral hemorrhage (ICH) demonstrated that administration of anti-HMGB1 mAb inhibited HMGB1, brain edema, microglial activation, mRNA expression of pro-inflammatory cytokines, and apoptotic cell death in peri-hematoma areas, leading to improved neurological performance and reduced plasma levels of HMGB1 [31]. Overall, HMGB1 exhibits strong pro-inflammatory properties primarily through its interaction with the receptor for advanced glycation end products (RAGE), making its blockade a potential therapeutic strategy for brain injury [83].

#### 2.3.2. Cluster of Differentiation 11/18 (CD11/CD18)

Cluster of differentiation 11/18 (CD11/CD18) integrins moderate the entry of leukocytes into the central nervous system. Previous research by Bao and colleagues utilized an anti-CD11d mAb to block the CD11d/CD18 and VCAM-1 interaction following experimental spinal cord injury in rats [32]. Their studies demonstrated that treatment with the CD11 mAb for up to 48 h after spinal cord injury enhanced functional recovery by decreasing the number of neutrophils and macrophages and preventing the formation of reactive free radicals, lipid peroxidation, protein nitration, and DNA damage [32,33,34,35,36]. Given that these mechanisms also contribute to secondary injury in TBI, researchers explored the potential therapeutic benefits of anti-CD11d for TBI. Their findings indicated that the antibody reduced neutrophil and macrophage infiltration within the injured brain, subsequently decreasing lipid peroxidation, free radical formation, astrocyte activation, amyloid precursor protein expression, and neuronal loss [84]. These effects corresponded with reduced impairments in tests of spatial cognition, anxiety, and sensorimotor function in the rats [84].

### 2.4. FDA-Approved mAbs

#### 2.4.1. Interleukin 6 and Interleukin 6 Receptor (IL-6, IL-6R)

IL-6 is a cytokine that exhibits both pro-inflammatory and anti-inflammatory properties. It is produced by various cell types in response to stimuli such as trauma and infection [85]. IL-6 exerts its effects by binding to its specific receptor, IL-6R, which exists in both soluble and membrane-bound forms. The binding of IL-6 to IL-6R occurs in conjunction with a transducer protein known as glycoprotein 130 (gp130). Notably, soluble IL-6R can also initiate signaling through a process called trans-signaling. Together, IL-6, IL-6R, and two gp130 molecules form a four-part complex at the cell surface, activating genes with IL-6 response elements via the JAK-STAT pathway. This activation commonly leads to the production of acute phase proteins [86], thereby promoting inflammation.

Currently, two IL-6R monoclonal antibodies (mAbs), Tocilizumab and Sarilumab, are FDA-approved for the treatment of rheumatoid arthritis (RA) [19]. IL-6 plays a crucial role in the pathogenesis of RA, producing extensive systemic effects. A comprehensive review by Narazaki and colleagues [87] highlights the multifaceted effects of IL-6 in RA. In summary, IL-6 influences the differentiation of T and B lymphocytes, vascular homeostasis, the acute phase response, and coagulability. It promotes the differentiation of plasma blasts into plasma cells, contributing to the hypergammaglobulinemia observed in RA. Additionally, IL-6 stimulates the differentiation of CD4+ T cells into Th17 cells, which negatively impacts T regulatory cell (Treg) differentiation. Furthermore, IL-6 enhances T follicular helper (Tfh) cell differentiation, promoting a T cell-dependent B-cell response. IL-6 also affects vascular homeostasis by increasing vessel permeability and influences the acute phase response by elevating levels of C-reactive protein, complement C3, fibrinogen, and thrombopoietin. Moreover, IL-6 contributes to a hypercoagulable state through two mechanisms: it increases thrombopoietin during the altered acute phase response, which enhances megakaryocyte production in the bone marrow and leads to thrombocytosis. Simultaneously, IL-6 upregulates the expression of tissue factor on monocytes. Tocilizumab and Sarilumab mitigate these effects by blocking both membrane-bound and soluble IL-6 receptors.

In contrast, a third FDA-approved mAb, Siltuximab, binds directly to IL-6, competitively inhibiting IL-6R stimulation and its downstream effects [37]. Siltuximab is indicated for Castleman’s disease (CD) but is also being investigated for various cancers, including ovarian, prostate, and lung cancers, as well as multiple myeloma. In CD, IL-6 inhibition primarily targets the aberrant secretion of IL-6 by germinal center B lymphocytes and plasma cells [38].

#### 2.4.2. Interleukin-1β (IL-1β)

IL-1β is a pro-inflammatory cytokine that requires interaction with the type I IL-1 receptor (IL-1RI) and the IL-1 receptor accessory protein (IL-1RAcP) to form a heterotrimeric complex. This complex brings together the intracellular Toll-IL-1 receptor domains, ultimately activating NF-κB and downstream transcription [39]. IL-1β stimulation results in both localized and systemic effects, playing a crucial role in resolving infections; however, chronic stimulation can be detrimental. In the context of TBI, IL-1β stimulation can lead to the recruitment of inflammatory cells to sites of inflammation, propagating the inflammatory response and inducing the production of reactive oxygen species through enzymes such as cyclooxygenase 2 and inducible nitric oxide synthase [88].

The FDA has approved Canakinumab for treating cryopyrin-associated disorders (CAP), including Muckle–Wells syndrome and familial cold auto-inflammatory syndrome. CAP arises from the uncontrolled over-secretion of IL-1β due to mutations in the cryopyrin-coding gene NLRP3. These mutations lead to functional changes in the inflammasome protein cryopyrin, which regulates IL-1β secretion [89]. Canakinumab is a human monoclonal antibody that binds free IL-1β, preventing its interaction with IL-1RI and IL-1RAcP, thereby inhibiting the formation of the signaling complex. This blockade reduces NF-κB activation and downstream pro-inflammatory transcription regulated through Toll-IL-1 receptor domains [39].

## 3. mAbs and Neurodegeneration

### 3.1. Pathophysiology Relevance

It is widely acknowledged that TBI is associated with increased risks of developing dementia, including Alzheimer’s disease (AD) and Parkinson’s disease (PD) [1,90,91,92,93,94]. Diffuse axonal injury is one of the most common neuropathological features of TBI, which is characterized by the mechanical deformation of axons. Its subsequent neuronal dysfunction includes abnormal glutamate release, axonal transport interruption, swelling, and accumulation of proteins, such as amyloid-beta (Aβ), phosphorylated tau (pTau), and α-synuclein [95,96,97,98]. These proteinopathies are also pathological hallmarks in Alzheimer’s (Aβ and pTau) [99,100,101,102] and Parkinson’s diseases (α-synuclein) [103,104,105]. Plaques composed of Aβ peptides are now recognized as a common pathology of both acute and chronic stages of TBI patients [94,98]. TBI-induced axonal damage leads to the accumulation of amyloid precursor protein (APP) and its cleavage enzymes, including beta secretase 1 and presenilin 1 in axonal swellings. Cleaved APP further forms pathogenic species of amyloid-beta, which are released into the brain and generate plaques within hours following TBI [97,106,107]. Toxic amyloid-beta plaques contribute directly to neuronal loss observed after TBI [97]. Widespread amyloid-beta plaques in some TBI patients persist for months or years and further trigger long-term neurodegenerative processes, which leads to progressive neurodegeneration [97,107,108,109].

In addition to amyloid-beta pathology, tau proteins are hyperphosphorylated and accumulated after TBI [94,97,107,109,110]. The microtubule-associated protein tau is a key constituent of axons and functions to stabilize microtubules, and it contributes to the regulation of axonal transport, neuronal development, postsynaptic scaffolding, and apoptosis [111,112,113]. In sports and military-related TBI patients and experimental animal models, mechanical axon deformation and impaired axonal transport during injury may induce tau disassociation from microtubules, which leads to tau hyperphosphorylation, misfolding, and aggregation. These events further produce a highly pathogenic tau species (cis-P-tau), which contributes to apoptosis, mitochondrial damage, and abnormal long-term potentiation, resulting in axonal damage and neuronal loss [40]. Interestingly, tau is also aberrantly acetylated in various neurodegenerative conditions, including AD and TBI [41].

TBI has long been associated with Parkinson’s disease; α-synuclein protein aggregation is a critical pathology of PD [114]. It is a presynaptic protein that plays an important role in synaptic vehicle recycling. In a chronic TBI rat model, dopaminergic neurons in the substantia nigra were significantly decreased. In parallel, an increased abnormal accumulation of α-synuclein was detected, which suggested that α-synuclein may function as a pathological link between the chronic effects of TBI and PD-like neurodegeneration [92,115].

### 3.2. Pre-Approval mAbs

Pre-clinical studies in mouse models of impact and blast TBI showed increased cis-P-tau levels and related axonal damage. Administration of anti-cis-tau monoclonal antibody (mAb) blocked cis-P-tau pathology and restored neuronal dysfunction. These results suggest that cis-tau mAbs can be used as a potential therapy for preventing the development of TBI-induced tauopathy [40]. Similarly, anti-acetylated-tau treatment reduces tau pathology, rescues glial responses, and improves neurobehavioral impairment [41].

Post-traumatic proteinopathies have similarities to neurodegenerative diseases, in particular AD [98,116]. mAb therapies targeting proteinopathies have been used for the treatment of AD and PD, which also provide a promising strategy for treating TBI. Several mAbs targeting amyloid-beta have been developed to treat AD, including Aducanumab, Remternetug, Donanemab (aka N3pG or Kisunla), Leqembi, and Solanezumab [45,46,47,117,118]. α-synuclein-targeted mAbs for PD were developed and used to improve α-synuclein-associated neurodegeneration [48,49,50,51].

### 3.3. FDA-Approved mAbs

Among these mAbs, Aducanumab was the first FDA-approved (accelerated approval) mAb targeting Aβ aggregates to treat AD [42]. It decreases amyloid-beta plaques, which is accompanied by a modest slowing of cognitive decline. Though post-approval phase 4 is needed to verify clinical benefit, it is a big step forward for treating and preventing AD. Leqembi was granted accelerated approval by the FDA in 2023, marking it as the second monoclonal antibody targeting Aβ approved for the treatment of AD. The pivotal Phase III clinical trial, CLARITY AD, evaluated the drug in 1795 patients exhibiting symptoms of mild dementia due to early-stage AD. Results from this trial demonstrated that after 18 months of treatment, the rate of decline in cognitive and memory function was reduced by 27% in participants receiving Leqembi compared with those given a placebo [43]. Donanemab is the third humanized IgG1 monoclonal antibody developed from mouse mE8-IgG2a. It recognizes Aβ (3–42), an aggregated form of Aβ found in amyloid plaques. Donanemab targets deposited plaque itself to clear existing amyloid burden from the brain rather than merely preventing the deposition of new plaques or the growth of existing plaques [44].

## 4. mAbs and Coagulopathy

### 4.1. Pathophysiology Relevance

Under physiologic conditions, the coagulation system is maintained in a dynamic equilibrium by a careful balance between coagulation cascades to form clots and fibrinolysis to break down clots. Hematologic aberrations are manifested by disruption of the balance between the coagulation system and the fibrinolytic system. TBI represents one of these disruptions; it causes early hypercoagulation that transitions to a subsequent hypocoagulable state and is known to correlate with worsened outcomes, including morbidity and mortality. The underlying mechanisms are poorly understood, but since the patients lack the traditional causes of coagulopathy, including significant blood loss or fluid administration, TBI-induced coagulopathy is believed to be pathogenic.

The BBB is the semi-permeable barrier of cerebral vasculature and is susceptible to shear forces during mechanical loading conditions. BBB compromise is well documented following TBI and can lead to extravasation of brain-derived molecules. Among these molecules are pro-coagulants and tissue factors that cause widespread activation of the extrinsic coagulation cascade upon reaching circulation [119]. The clotting process via the extrinsic pathway involves the production of Factor VIIa/TF complex, which generates Factor Xa. Factor Xa mediates the cleavage of prothrombin into thrombin that, in turn, acts as a protease catalyzing the conversion of soluble fibrinogen into insoluble strands of fibrin, forming a blood clot. In addition, thrombin activates platelets and Factors V and VIII, stimulating more thrombosis [120]. TBI-induced endothelial damage, including glycocalyx degradation, further accelerates coagulation through direct interaction between the circulating components of blood and the endothelial wall. This hypercoagulable state confers additional risk to TBI patients by augmenting the risk of microthrombi formation and subsequent ischemic injury [119]. In fact, TBI patients face a much greater risk of developing embolic and thrombotic complications, including deep vein thrombosis and pulmonary embolism.

Hypercoagulation causes the eventual depletion of clotting factors and platelets, likely causing the transition to a hypocoagulable state [121]. This transition has been observed clinically by prolonged prothrombin and partial thromboplastin times seen hours after injury. In addition, a clinical study reported that the level of fibrin degradation products reached a peak at 6 h post-TBI and then returned to normal within 24 h, indicating acute and transient hyperfibrinolysis. The hypocoagulation and hyperfibrinolytic state creates a particular vulnerability of the brain to bleeding diathesis and progression of intracerebral hemorrhage [3,120]. Importantly, the hyperfibrinolysis and increased fibrin degradation products were positively correlated with poor clinical outcome after TBI.

### 4.2. FDA-Approved mAbs

mAb therapies have proven successful in influencing several aspects of the coagulation process, including clot prevention, formation, and clearance. Drugs, including Abciximab [52] and Caplacizumab [53], have been approved by the FDA for use in individuals undergoing coronary artery procedures and thrombotic thrombocytopenic purpura, respectively. These drugs effectively target the glycoprotein IIb/IIa–von Willebrand factor interactions to prevent platelet aggregation and elicit anti-coagulation effects. Abciximab binds directly to glycoprotein IIb/IIIa surface receptor, which plays a critical role in the aggregation of platelets. Through steric hindrance and conformational changes, the binding of Abciximab effectively blocks fibrinogen, von Willebrand factor, and other adhesion molecules from accessing the receptor sites necessary to initiate platelet aggregation. On the other hand, Caplacizumab is able to achieve a similar effect by inhibiting the other side of the interaction. Caplacizumab is specific to von Willebrand factor, thereby compromising its ability to interact with platelets and begin the aggregation process.

## 5. mAbs and Vascular Function

### 5.1. Pathophysiology Relevance

In the normal brain, the BBB tightly regulates CNS homeostasis, which is critical for neuronal function and restricts the entry of blood-borne factors and circulating immune cells. It is composed of cerebrovascular endothelial cells (ECs) joined by tight junctions and adherens junctions. Glial cells, such as astrocytes and microglia, physically interact with ECs, which are also critical to BBB and EC integrity [122,123,124,125]. Shortly after TBI, the cerebral vasculature is damaged in the form of mechanical disruption and increased permeability of the BBB, which contributes to the pathogenesis of TBI and can lead to secondary injury, including hemorrhage, ischemia, vasogenic edema, and BBB dysfunction. BBB disruption is often associated with poor long-term outcomes and further worsens the secondary injury [74,125,126].

One major indicator of BBB disruption is the activation of ECs. In response to a surge of pro-inflammatory cytokines and increased chemokines after TBI, endothelial cells are activated, which further induces the surface expression of a number of cellular mediators [74,125]. Among these mediators are cell adhesion molecules, including selectins, integrins, intercellular adhesion molecules (ICAM-1 or CD54), and vascular cell adhesion molecules (VCAM-1 or CD106). Cell adhesion molecules are involved in the adhesion of leukocytes and immune cells to the endothelial wall and mediate leukocyte transmigration across the BBB into brain parenchyma [127,128,129]. During TBI, the endothelial wall is destroyed by the adhesion of leukocytes and platelets. Infiltrating leukocytes further drive the neuroinflammatory response and exacerbate secondary brain injury through the production of pro-inflammatory mediators, free radicals, and oxidative stress [74,125,127]. The selectin family of adhesion molecules, such as P-selectin, is not only expressed in endothelial cells but also in platelets [130]. In addition to promoting leukocyte infiltration into injured brain regions during TBI-induced inflammation, P-selectin also mediates platelet–platelet interactions. It facilitates platelet aggregation and platelet–leukocyte interactions, both important processes in the development of thrombosis [130]. Elevated P-selectin in TBI patients promotes multicellular aggregation in the bloodstream, which results in vaso-occlusion, further reduces blood flow, and increases the risk of brain ischemia [131].

In addition to the induction of cell adhesion molecules, numerous vascular-associated signaling molecules, including vascular endothelial growth factors (VEGFs), are elevated after TBI [74,125,132]. Among these VEGFs, VEGF-A is a potent mitogen for vascular endothelial cells, which is not only critical for vasculogenesis during embryonic development but is also a vital factor for physiological angiogenesis associated with wound healing, ischemia, and tumorigenesis at the adult stage [133]. VEGF-A is mediated by VEGF receptor tyrosine kinases, including VEGFR-1 and VEGFR-2 [133,134,135]. TBI-induced VEGF further promotes angiogenesis and vasculature repair in the damaged brain through interaction with its receptor. VEGF-A is also known as vascular permeability factor. In cultured brain and retinal endothelial cells exposed to VEGF-A, VEGF-A was found to downregulate the expression of tight junction proteins, including occludin and claudin5, which resulted in the increased permeability of EC monolayers and vascular leakage, thus promoting the development of edema [136,137,138].

### 5.2. FDA-Approved mAbs

mAb therapies targeting functions of cell adhesion molecules and vascular signaling molecules have been effectively used for the treatment of several disease types, including sickle cell disease, colorectal cancer, gastric cancer, and macular degeneration. Among these FDA-approved mAbs, Crizanlizuman is designed to target P-selectin to prevent vaso-occlusion in patients with sickle cell disease [54]. Bevacizumab (Avastin) is an anti-angiogenic drug that is used to treat colorectal cancer. It targets VEGF-A and prevents the binding of VEGF-A to its receptors. Thus, it inhibits tumor vessel growth [55]. In addition, Bevacizumab treatment showed sex-specific effects in a rat mTBI model [139]. Ramucirumab is another anti-angiogenic drug and has been used to treat patients with gastric cancer. It blocks the activation of VEGFR-2, the primary receptor for VEGF-A, to reduce tumor angiogenesis involved in the development and progression of gastric cancer [57]. Brolucizumab is used to treat patients with macular degeneration. They are designed to bind to and block VEGF-A. By blocking VEGF-A, these drugs reduce macular edema caused by vascular leakage [56].

## 6. mAbs and Excitotoxicity

Glutamate is a key excitatory neurotransmitter that mediates many processes, including neural signaling in synapses, learning, and memory. High concentrations of glutamate are harmful. Excitotoxicity occurs when neurons are subjected to excessive stimulation by glutamate, leading to the continuous activation of NMDA (N-methyl-D-aspartate) receptors [140]. These receptors consist of multiple subunits, with GluN1 being one of them. Excitotoxicity is a pathological process that occurs after TBI, resulting in neuronal damage and cell death. During TBI, the brain experiences a heightened energy demand due to both primary and secondary injuries, which increases the need for glycolysis and subsequently elevates glucose supply, leading to overstimulation [141]. Uncontrolled release of glutamate triggers calcium influx into the cytoplasm, activating damaging signaling pathways that can result in apoptosis [142]. Consequently, blocking an NMDA receptor subunit appears to be a promising strategy for preventing excitotoxicity in TBI. Excitotoxicity is not only significant in TBI pathology but also contributes to the development of multiple sclerosis (MS) by damaging oligodendrocytes [58]. Thus, targeting NMDA receptors in an MS model may also yield beneficial outcomes. The interaction between tissue plasminogen activator (tPA) and GluN1 has been shown to promote excitotoxicity [143].

### Pre-Approval mAbs

In a recent study using an experimental autoimmune encephalomyelitis (EAE) model in mice [144], the researchers used an anti-NMDAR mAb called Glunomab to block the interaction of tPA and GLUN1. The researchers employed an anti-NMDAR monoclonal antibody called Glunomab to block the interaction between tPA and GluN1. The results indicated that Glunomab effectively mitigated neurological impairments, which correlated with the preservation of the blood–brain–spinal cord barrier and reduced leukocyte infiltration. Another study [145] utilized a thromboembolic stroke model in mice to evaluate a polyclonal antibody designed to prevent the interaction between tPA and the NR1 subunit of NMDA receptors, aiming to counteract excitotoxic effects. This polyclonal antibody specifically targeted tPA receptors and successfully inhibited rtPA from increasing NMDA-induced calcium release in cortical neurons. Ultimately, it demonstrated significant neuroprotective effects from 20 min to 4 h post-clot without altering NMDA neurotransmission and improved long-term neurological outcomes [145].

## 7. mAbs and Oxidative Stress

TBI-induced oxidative stress results from the excessive production of free radicals, including reactive species of oxygen and nitrogen (ROS and RNS), which overwhelm endogenous antioxidant systems [146,147]. These free radical species are generated after TBI from various sources, such as dysfunctional mitochondria and impaired metabolism, as well as excitotoxic and inflammatory pathway activation [147]. They alter the physiology of lipids, proteins, and DNA, which causes dysfunction or loss of activity and eventually leads to cell apoptosis, necrosis, and neuronal damage. Free radicals react with the lipids on cell/neuronal membranes, causing lipid peroxidative damage. This alters cell membrane fluidity, increases membrane permeability, decreases membrane ATPase activity, and disrupts the cellular/neuronal structure and integrity [148]. These ROS-induced alterations further contribute to TBI pathology and, ultimately, neurodegeneration. Antioxidant drugs targeting ROS/RNS have been used before or after TBI to mitigate oxidative injury in animal models and showed beneficial effects. However, most of them have failed in clinical trials [146,147].

### Pre-Approval mAbs

There have been few studies targeting the post-traumatic oxidative environment. However, mAbs targeting MMP-9 have been tested in several rodent models, such as focal cerebral ischemia and intestinal fibrosis. MMP-9 cleaves endothelial basal lamina and the tight junction proteins of the BBB under normal conditions but has also been associated with oxidative stress by several studies [149,150,151,152,153,154,155]. In a rat model of focal cerebral ischemia, MMP-9 expression is increased in endothelial cells and infiltrating neutrophils after focal ischemia. Systemically, administration of the anti-MMP-9 mAb significantly reduced the infarct size, thus mitigating brain injury [59]. Similarly, increased serum MMP-9 levels have been observed in a mouse model of intestinal fibrosis, which is highly associated with the pathology. The anti-MMP-9 treatment reduced collagen deposition and hydroxyproline content in intestinal grafts, indicating reduced fibrosis [60]. These data indicate the efficacy of anti-MMP-9 mAbs in these animal models. It also suggests that selective MMP-9 inhibition using mAbs is a promising therapeutic strategy for the treatment of MMP-9-regulated processes, including TBI-associated oxidative stress and BBB dysfunction. Interestingly, andecaliximab against MMP-9 is currently being examined with a combination chemotherapy regimen in a Phase III study with gastric and gastroesophageal junction adenocarcinoma and demonstrates encouraging beneficial effects without added toxicity [61].

## 8. mAbs and Safety

As with most therapeutic interventions, mAb treatment is not without risk of adverse reactions/side effects. mAb therapy can elicit a diverse range of adverse effects, which are discussed and summarized thoroughly by Hansel et al. [156]. Some of the most common side effects include immune reactions, infections, platelet and thrombotic disorders, organ-specific toxicity, anaphylactic (IgE-mediated) reactions, serum sickness syndrome, cytokine storm, autoimmune diseases, and cancer [156,157]. The previous clinical testing of mAbs may provide some guidance for what to expect when testing and developing mAb therapies. However, it should be noted that the specificity of mAbs in biological systems may make them likely to yield specific and new reactions. TBI pathology is known to alter the metabolism and clearance of therapeutics, which introduces another important variable [158,159]. These adverse effects could be minimized by sound pre-clinical and clinical practice. As such, it is important that future studies evaluating the efficacy of mAbs for TBI treatment consider the whole-body safety effects as well. The development and validation of proper in vitro safety tests during pre-clinical study and the advancement of mAbs, such as the production of next-generation antibodies like bispecific antibodies, trispecific antibodies, and low molecular weight antibodies, may also facilitate the improvement of the safety of mAbs [156,160].

## 9. Conclusions

TBI pathophysiology is complex and multifaceted, often involving inflammation, vascular function, coagulopathy, oxidative stress, and neurodegeneration. Many of the pathological processes are conserved within other diseases, which may confer important observations in considering treating TBI. Historically, clinical heterogeneity and lack of robust and specific treatment effects have prevented the successful development of treatment strategies for TBI. However, recent advancements in mAb technology have demonstrated success in complex neurological diseases because of their specificity in action and fewer adverse effects as compared to existing treatment options. Herein, we have explored how the current pre-clinical and clinical mAb therapies may harbor potential for the treatment of TBI through targeting specific pathological processes. As ongoing studies continue to better define the molecular underpinnings of TBI pathology and secondary injury mechanisms, the identification of novel therapeutic targets will continue. Therefore, it is imperative that we begin to consider how we can target them with therapeutic strategies, including mAbs.

Considering numerous molecular mechanisms that contribute to the complexity of TBI, future efforts for mAb therapies could target bispecific mAbs or mAb mixtures that control multiple molecular pathways in TBI. In addition, one of the limitations of therapeutic mAbs is their inability to cross the blood–brain barrier, which prevents them from reaching their target in the brain at the therapeutic level to be effective. To improve the bioavailability of mAbs in the brain, many approaches for delivery have been explored with success, including extracellular vesicles, engineered bispecific mAbs, and nanoparticles. The continuous development of brain-penetrant mAbs could be beneficial, particularly for TBI. These mAb-based therapeutics will likely facilitate drug therapy in the TBI research field and will hopefully be able to address unmet medical needs for TBI patients.

## Figures and Tables

**Table 1:** Pre-clinical mAbs and FDA-approved mAbs targeting secondary injury pathology in TBI.

mAb	Targeted Secondary Injury Mechanism	Stage of Development	Effects/Indications	Reference
HMGB1	Inflammation	Pre-clinical	Reduce microglial activation and neuronal death	[30,31]
CD11/CD18	Inflammation	Pre-clinical	Reduce brain edema and microglial activation	[32,33,34,35,36]
Tocilizumab, Sarilumab Siltuximab	Inflammation (IL-6/IL-6R)	FDA-approved	Rheumatoid arthritis Castleman’s disease; ovarian, prostate, and lung cancers Multiple myeloma	[19] [37,38]
Canakinumab	Inflammation (IL-1β)	FDA-approved	Cryopyrin-associated disorders	[39]
Cis-P-tau	Neurodegeneration (P-tau)	Pre-clinical	Block cis-P-tau pathology and restore neuronal dysfunction	[40]
Acetylated-tau	Neurodegeneration (acetylated-tau)	Pre-clinical	Reduce tau pathology and glia activation Improve neurobehavioral impairment	[41]
Aducanumab Leqembi Donanemab	Neurodegeneration (amyloid-beta)	FDA-approved	Alzheimer’s disease	[42] [43] [44]
Remternetug	Neurodegeneration (amyloid-beta)	Phase III (Clinical Trial #:NCT05463731)	Alzheimer’s disease	[45,46,47]
α-synuclein	Neurodegeneration	Pre-clinical	Parkinson’s disease	[48,49,50,51]
Abciximab	Coagulopathy (glycoprotein IIb/IIIa)	FDA-approved	Coronary artery procedures	[52]
Caplacizumab	Coagulopathy (glycoprotein IIb/IIIa)	FDA-approved	Thrombotic thrombocytopenic purpura	[53]
Crizanlizuman	Vascular function (P-selectin)	FDA-approved	Sickle cell disease	[54]
Bevacizumab Brolucizumab	Vascular function (VEGF-A)	FDA-approved	Colorectal cancer Macular edema	[55] [56]
Ramucirumab	Vascular function (VEGFR)	FDA-approved	Gastric cancer	[57]
NMDA receptor	Excitotoxicity	Pre-clinical	Multiple sclerosis	[58]
MMP-9	Oxidative stress	Pre-clinical	Reduce fibrosis, oxidative stress, and BBB dysfunction	[59,60]
Andecaliximab	Oxidative stress (MMP-9)	Phase III (Clinical Trial #: NCT02545504)	Gastric cancer	[61]
